# Does the adoption of EUCAST susceptibility breakpoints affect the selection of antimicrobials to treat acute community-acquired respiratory tract infections?

**DOI:** 10.1186/1471-2334-12-181

**Published:** 2012-08-06

**Authors:** Anna Marchese, Susanna Esposito, Ramona Barbieri, Matteo Bassetti, Eugenio Debbia

**Affiliations:** 1 Microbiology Unit, DISC Department, University of Genoa, R. Benzi 10, 16132, Genoa, Italy; 2 Department of Maternal and Pediatric Sciences, University of Milan, Fondazione IRCCS Cà Granda Ospedale Maggiore Policlinico, Milan, Italy; 3 Infectious Diseases Division, S. Maria della Misericordia University Hospital, Udine, Italy

**Keywords:** CLSI, Interpretive criteria, Resistance, Antibiotics, *S. pneumoniae*, *H. influenzae*, *S. aureus*, *M. catarrhalis*

## Abstract

**Background:**

In several European Countries, by the end of 2012, CLSI guidelines will be replaced by EUCAST. We compared antimicrobial susceptibility results of a large number of respiratory pathogens using both EUCAST and previously adopted CLSI criteria to evaluate the impact on susceptibility patterns and the possible consequences that could occur in clinical practice due to this replacement.

For *S. pyogenes* and *S. aureus*, the interpretation of susceptibility data using the EUCAST criteria did not produce relevant changes in comparison to CLSI.

Against *S. pneumoniae*, more restrictive EUCAST breakpoints could lead to increased benzylpenicillin and/or amoxicillin-clavulanate resistance rates, which in turn could translate in increased dosages of these antibiotics or usage of alternative agents for respiratory tract infections.

Against *S. pneumoniae, M. catarrhalis* and *H. influenzae*, cefuroxime-axetil and cefaclor produced the most divergent results depending on the breakpoints adopted and these striking differences could lead to the revision of those guidelines suggesting these two cephalosporins as alternatives in the management of upper respiratory tract infections.

**Discussion:**

Many differences exist between CLSI and EUCAST breakpoints. However, only in a few cases do these differences translate in major interpretive category discrepancies. In countries adopting more restrictive EUCAST breakpoints, clinicians should be aware of these discrepancies and that they could be faced with antibiotic-resistant respiratory pathogens more frequently than before.

**Summary:**

The interpretive discrepancies between EUCAST and CLSI suggest that the discussion on the management of community-acquired respiratory tract infections is still open and further studies are desirable to better define the role of some antibiotics.

## Background

Acute community-acquired respiratory tract infections, including otitis media, sinusitis, exacerbations of chronic bronchitis, and community-acquired pneumonia, represent some of the most common infections treated by physicians [1].

The overall aetiology has not changed in recent years: *Streptococcus pyogenes, S. pneumoniaeHaemophilus influenzae* and *Moraxella catarrhalis* are the most common pathogens. Infection with ‘atypical pathogens’ such as *Mycoplasma pneumoniaeChlamydiophila pneumoniae* and *Legionella pneumophila* have also been reported [2,3]. As conjugate vaccines are introduced routinely, such as those against *H. influenzae* Type B and pneumococcus, they could change the aetiology of pneumonia, with “atypical pathogens” likely to become proportionally more important. In the community the management of these conditions is generally empirical [4-7] and recommendations on the most appropriate first-line agents should also be based on updated local epidemiological data derived by the availability of antimicrobial susceptibility testing results, which in turn depend on interpretative criteria or ‘breakpoints’ used to divide a bacterial population into susceptible, intermediate and resistant categories.

Because different agencies such as the CLSI (Clinical and Laboratory Standards Institute) and EUCAST (European Committee on Antimicrobial Susceptibility Testing) may suggest different breakpoints, the assignment of a pathogen to a defined category (susceptible, intermediate and resistant) depends on the specific guideline adopted. As a consequence, breakpoint discrepancies could have an important impact, possibly leading to divergent conclusions impinging on the selection of the drug to be used by the physician.

Since in several European Countries, CLSI guidelines have been or are going to be replaced by EUCAST guidelines, we compared antimicrobial susceptibility results reported in literature on a large number of respiratory strains (mainly available at the EUCAST website) [8] using both EUCAST and previously adopted CLSI breakpoints to evaluate the impact of breakpoint changes on the overall susceptibility patterns and the possible consequences occurring in clinical practice.

## Methods

Antimicrobial Susceptibility data (minimal inhibitory concentration distributions) obtained mainly from the EUCAST website [8] and from two published articles [9,10] were analysed using the latest available version of CLSI (Twenty-second Informational Supplement M100-S22 and Methods for Antimicrobial Dilution and Disk Susceptibiltiy Testing of Infrequently Isolated or Fastidious Bacteria; Approved Guideline- Second Edition- M45-A2) [11,12] and EUCAST documents [13] breakpoints and were compared.

It is worth noting that the percentages of susceptibility obtained cannot be used to assess susceptibility rates in any epidemiological context since, as already stated in the EUCAST website, susceptibility data were aggregated from different sources, countries and time periods.

Only those antibiotic/pathogen combinations, for which both EUCAST and CLSI provide breakpoints, were included in the study, with the exception of *S. pyogenes*/chloramphenicol and *S. aureus*/netilmicin, due to the very limited number of minimal inhibitory concentration (MIC) values available for these two combinations. When, for each bacterial species/antibiotic combination, the percentages of susceptibility calculated by the two interpretive criteria (CLSI and EUCAST) differed by 0- < 1%, the results were considered comparable.

Discrepancies were arbitrarily defined as follows: minor discrepancies as differences ranging between 1 and 10% (1- < 10%), major discrepancies as differences ranging between 10 and 25% (10- < 25%) and very major discrepancies as differences greater than or equal to 25% (≥25%).

## Results

Table 1 reports the antimicrobial susceptibilities of *S. pyogenes* isolates to six antimicrobial agents, according to the available CLSI and EUCAST interpretive breakpoints.

**Table 1:** **Antimicrobial susceptibility of* S. pyogenes*as classified by CLSI and EUCAST §**

**Antimicrobial agent**^**a**^**(n° of strains)**	**CLSI susceptibility breakpoint (mg/L)**	**EUCAST susceptibility breakpoint (mg/L)**	**CLSI %S**	**EUCAST %S**	**Type of discrepancy**^**b**^
Penicillin (934)	**≤**0.12	**≤**0.25	99.9	100	-
Azithromycin (22.884)	**≤**0.5	**≤**0.25	91.7	91.2	-
Clindamycin (10.994)	**≤**0.25	**≤**0.5	96.6	96.7	-
Levofloxacin (26.775)	**≤**2	**≤**1	99.0	93.1	minor
Vancomycin (10.728)	**≤**1	**≤**2	100	100	-
Tetracycline (2.413)	**≤**2	**≤**1	83.9	83.7	-

§ CLSI [11] and EUCAST [13].

^**A**^For Erythromycin, Clarithromycin, Linezolid and Daptomycin CLSI and EUCAST suggested the same susceptibility breakpoints.

^**b**^ Discrepancy as defined in the materials and methods section.

Globally, the susceptibility rates calculated following the two guidelines appear to be identical or very similar (<1%), with the exception of levofloxacin. Using the CLSI breakpoint, the percentage of levofloxacin susceptible isolates was lower than that obtained following the EUCAST criteria (99.0% *vs* 93.1% respectively), leading to a minor discrepancy.

Comparable results or minor discrepancies were observed for the great majority of antibiotics studied against *S. pneumoniae* (Table 2). However, adoption of the more restrictive EUCAST breakpoints for cefaclor and ofloxacin produced very major discrepancies. Using the EUCAST breakpoints, the percentage of cefaclor and ofloxacin susceptible strains was drastically reduced (<1%) in comparison to CLSI results (>90% of susceptibile strains). For other beta-lactams the adoption of EUCAST breakpoints caused a reduction in the percentages of susceptible strains leading to minor (penicillin, cefpodoxime, cefuroxime axetil, cefotaxime, ceftriaxone, meropenem and imipenem) and major discrepancies (amoxicillin-clavulanate), when compared with CLSI results.

**Table 2:** **Antimicrobial susceptibility of* S. pneumoniae*as classified by CLSI and EUCAST §**

**Antimicrobial agent**^**a**^**(n° of strains)**	**CLSI susceptibility breakpoint (mg/L)**	**EUCAST susceptibility breakpoint(mg/L)**	**CLSI %S**	**EUCAST %S**	**Type of discrepancy**^**b**^
Benzyl-penicillin (37.642)	**≤**2*^c^	**≤**1**	94.2	87.4	minor
Amoxicillin-clavulanate (2725)	**≤**2	**≤**0.5***	99.2	87.6	major
Cefaclor (2.581)	**≤**1	**≤**0.032	92.3	0.5	very major
Cefpodoxime (5.725)	**≤**0.5	**≤**0.25	84.1	82.5	minor
Cefuroxime axetil (32.729)	**≤**1	**≤**0.25	79.6	73.6	minor
Cefotaxime (12.800)	**≤**1^c^	**≤**0.5	99.3	97.6	minor
Ceftriaxone (3.138)	**≤**1^c^	**≤**0.5	98.4	89.7	minor
Meropenem (575)	**≤**0.25^c^	**≤**2	97.7	99.8	minor
Ertapenem (3.680)	**≤**1	**≤**0.5	99.7	99.0	-
Imipenem (1.643)	**≤**0.12	**≤**2	95.4	99.9	minor
Moxifloxacin (26.746)	**≤**1	**≤**0.5	98.5	98.4	-
Ofloxacin (4.412)	**≤**2	**≤**0.125	95.4	0.0	very major
Azithromycin (63.481)	**≤**0.5	**≤**0.25	69.1	68.8	-
Telithromycin (7.034)	**≤**1	**≤**0.25	99.4	95.9	minor
Clindamycin (38.126)	**≤**0.25	**≤**0.5	81.4	81.6	-
Vancomycin (51.053)	**≤**1	**≤**2	99.8	100	-

§ CLSI [11] and EUCAST [13].

^**a**^For Cefuroxime parenteral, Levofloxacin, Erythromycin and Clarithromycin, CLSI and EUCAST suggested the same susceptibility breakpoints.

^**b**^Discrepancy as defined in the materials and methods section.

^**c**^Breakpoint refers to non meningeal infections.

* breakpoint refers to a dosage of 2 million units 6 times daily.

** breakpoint refers to a dosage of 2.4 gm 4 times or 1.2 gm 6 times daily.

*** breakpoint refers to Ampicillin. The rate of susceptibiliy to Amoxicillin-clavulanate has been inferred from the rate of susceptibility to Ampicillin.

With regard to *H. influenzae,* no relevant differences were observed for 7 out of the 21 compared antimicrobial agents (Table 3). Among the beta-lactams, oral cephalosporins, cefaclor and cefuroxime-axetil were those producing the most divergent results depending on the breakpoints adopted. Similarly, for azithromycin clarithromycin and telithromycin, while high percentages of susceptible isolates (>99%, 82.9% and >98% respectively) were obtained when adopting CLSI breakpoints, less than 2% of the strains were susceptible when results were interpreted according to EUCAST breakpoints).

**Table 3:** **Antimicrobial susceptibility of* H. influenzae*as classified by CLSI and EUCAST §**

**Antimicrobial agent**^**a**^**(n° of strains)**	**CLSI susceptibility breakpoint (mg/L)**	**EUCAST susceptibility breakpoint(mg/L)**	**CLSI %S**	**EUCAST %S**	**Type of discrepancy**^**b**^
Ampicillin-sulbactam (223)	**≤**2	**≤**1	98.2	92.4	minor
Amoxicillin-clavulanate (47.030)	**≤**4	≤2	99.7	98.4	minor
Cefaclor (28.338)	**≤**8	**≤**0.5	92.6	3.5	very major
Cefixime (7.403)	**≤**1	**≤**0.125	99.9	97.9	minor
Cefpodoxime (20.842)	**≤**2	**≤**0.25	99.9	96.7	minor
Cefuroxime axetil (94.671)	**≤**4	**≤**0.125	97.9	1.3	very major
Cefuroxime parenteral (94.671)	**≤**4	**≤**1	97.9	76.9	major
Cefotaxime (13.655)	**≤**2	**≤**0.125	99.6	99.7	-
Ceftriaxone (170)	**≤**2	**≤**0.125	100	96.5	minor
Cefepime (396)	**≤**2	**≤**0.25	100	91.7	minor
Ceftibuten (444)	**≤**2	**≤**1	98.4	97.1	minor
Meropenem non meningitis (6.511)	**≤**0.5	**≤**2	99.9	100	-
Imipenem (3.828)	**≤**4	**≤**2	98.9	97.4	minor
Ciprofloxacin (12.794)	**≤**1	**≤**0.5	99.7	99.6	-
Levofloxacin (22.880)	**≤**2	**≤**1	99.9	99.8	-
Moxifloxacin (14.177)	**≤**1	**≤**0.5	99.7	99.8	-
Ofloxacin (3.762)	**≤**2	**≤**0.5	100	99.9	-
Azithromycin (29.942)	**≤**4	**≤**0.125	99.3	1.2	very major
Clarithromycin (27.816)	**≤**8	**≤**1	82.9	1.5	very major
Telithromycin (5.382)	**≤**4	**≤**0.125	99.0	0.5	very major
Tetracycline (39.928)	**≤**2	**≤**1	97.7	96.8	-

§ CLSI [11] and EUCAST [13].

^**a**^For Ampicillin, Chloramphenicol, Ertapenem, Rifampin and Trimethoprim/sulfamethoxazole CLSI and EUCAST suggested the same susceptibility breakpoints.

^**b**^ Discrepancy as defined in the materials and methods section.

Concerning *M. catarrhalis,* very major discrepancies, due to the adoption of different breakpoints on calculated susceptibility percentages, were only seen with cefaclor and cefuroxime-axetil (Table 4). Cefaclor and cefuroxime-axetil susceptibility percentages were dramatically reduced using EUCAST breakpoints (<2% *vs* 95.1% and 98.7% of susceptible strains when adopting CLSI criteria).

**Table 4:** **Antimicrobial susceptibility of* M. catarrhalis*as classified by CLSI and EUCAST §**

**Antimicrobial agent**^**a**^**(n° of strains)**	**CLSI susceptibility breakpoint (mg/L)**	**EUCAST susceptibility breakpoint(mg/L)**	**CLSI %S**	**EUCAST %S**	**Type of discrepancy**^**b**^
Amoxicillin-clavulanate (3.549)	**≤**4	**≤**1	100	99.9	-
Cefaclor (7.536)	**≤**8	**≤**0.12	95.1	0.27	very major
Cefuroxime axetil (15.381)	**≤**4	**≤**0.125	98.7	1.2	very major
Cefotaxime (2.737)	**≤**2	**≤**1	99.9	99.6	-
Ceftriaxone (5.187)	**≤**2	**≤**1	99.9	99.1	-
Clarithromycin (910)	**≤**1	**≤**0.25	100	99.9	-
Erythromycin (3.038)	**≤**2	**≤**0.25	100	99.7	-
Ciprofloxacin (11.119)	**≤**1	**≤**0.5	99.9	99.9	-
Levofloxacin (5.239)	**≤**2	**≤**1	100	99.9	-
Tetracycline (8.660)	**≤**2	**≤**1	97.7	94.8	-

§ CLSI [11] and EUCAST [13].

^**a**^For Chloramphenicol and Trimethoprim/sulfamethoxazole CLSI and EUCAST suggested the same susceptibility breakpoints.

^**b**^ Discrepancy as defined in the materials and methods section.

Regarding *S. aureus,* only minor discrepancies were detected between the susceptibility rates calculated using EUCAST and CLSI breakpoints for all compared antibiotics (Table 5).

**Table 5:** **Antimicrobial susceptibility of* S. aureus*as classified by CLSI and EUCAST §**

**Antimicrobial agent**^**a**^**(n° of strains)**	**CLSI susceptibility breakpoint (mg/L)**	**EUCAST susceptibility breakpoint(mg/L)**	**CLSI %S**	**EUCAST %S**	**Type of discrepancy**^**b**^
Teicoplanin (56.399)	**≤**8	**≤**2	99.9	98.4	minor
Gentamicin (45.807)	**≤**4	**≤**1	93.6	89.1	minor
Amikacin (6.446)	**≤**16	**≤**8	97.6	92.6	minor
Tobramycin (3.155)	**≤**4	**≤**1	94.7	88.2	minor
Azithromycin (7.223)	**≤**2	**≤**1	58.6	56.2	minor
Clarithromycin (7.146)	**≤**2	**≤**1	58.8	58.5	-
Erytromycin (36.118)	**≤**0.5	**≤**2	74.8	77.4	minor
Tetracycline (1.864)	**≤**4	**≤**1	74.6	74.3	-
Doxycycline (5.037)	**≤**4	**≤**1	97.6	88.7	minor
Minocycline (1.417)	**≤**4	**≤**0.5	99.4	96.9	minor
Clindamycin (25.879)	**≤**0.5	**≤**0.25	87.5	87.2	-
Trimethoprim (449)	**≤**8	**≤**2	91.5	89.1	minor
Rifampin (1.154)	**≤**1	**≤**0.064	96.4	95.0	minor

§ CLSI [11] and EUCAST [13].

^**a**^ For Penicillin, Oxacillin, Vancomycin, Daptomycin, Ciprofloxacin, Levofloxacin, Ofloxacin, Moxifloxacin, Chloramphenicol, Trimethoprim-sulfamethoxazole, Quinupristin-dalfopristin and Linezolid CLSI and EUCAST suggested the same susceptibility breakpoints.

^**b**^ Discrepancy as defined in the materials and methods section.

Major categorical shift, percentages of intermediate and resistant strains for *S. pneumoniae*, *H. influenzae* and *M. catarrhalis* are detailed in Table 6.

**Table 6:** Pathogen/antibiotic combinations that present major changes and potential therapeutic consequences

**Pathogen/antibiotic**	**CLSI**	**CLSI**	**CLSI**	**EUCAST**	**EUCAST**	**EUCAST**	**Major categorical shift**	**Potential therapeutic consequences**	**Possible alternative/s**
	**%S**	**% I**	**% R**	**%S**	**% I**	**% R**			
**S. pneumoniae/**									
Amoxicillin-clavulanate	95.6	4.3	0.1	73.4	13.9	12.7	S→I/R	Reduced or no efficacy	Increased dosage of amoxicillin-clavulanate
	(2457)	(110)	(2)	(1884)	(358)	(326)			
(2568)									
Cefaclor	92.3	1.7	5.9	0.5	86.7	13	S→I/R	Reduced or no efficacy	Other oral cephalosporin/high dosage of amoxicillin-clavulanate
(2581)	(2384)	(45)	(152)	(13)	(2238)	(330)			
Ofloxacin	95.4	4.3	0.3	0.0	99.5	0.3	S→I	Reduced efficacy	Fluoroquinolones (moxifloxacin, levofloxacin, gemifloxacin)
(4412)	(4208)	(190)	(14)		(4388)	(14)			
**H. influenzae/**									
Cefaclor	92.6	5.2	2.3	3.5	/	96.5	S→R	No efficacy	Other cephalosporin/amoxicillin-clavulanate
(28338)	(26337)	(1481)	(654)	(990)		(27482)			
Cefuroxime-axetil	97.9	1.5	0.6	1.3	75.6	23.1	S→I/R	Reduced or no efficacy	Other cephalosporin/amoxicillin-clavulanate
(94671)	(92668)	(1390)	(613)	(1249)	(71574)	(21848)			
Cefuroxime-	97.9	1.5	0.6	76.9	10.5	12.6	S→ I/R	Reduced or no efficacy	Other cephalosporin/amoxicillin-clavulanate
parenteral	(92668)	(1390)	(613)	(72823)	(9963)	(11885)				
(94671)										
Azithromycin	99.3	/	/	1.2	98.1	0.7	S→I	Reduced or no efficacy	Betalactam/fluoroquinolones	
(29942)	(29733)			(350)	(29383)	(209)				
Clarithromycin	82.9	15.3	1.8	1.5	98.1	0.3	S→I	Reduced or no efficacy	Betalactam/fluoroquinolones	
(27816)	(23045)	(4257)	(514)	(424)	(27296)	(96)				
Telithromycin	89.9	9.0	1.1	0.5	99.1	0.4	S→I	Reduced or no efficacy	Betalactam/fluoroquinolones	
(5382)	(4837)	(486)	(59)	(26)	(5335)	(21)				
**M. catarrhalis/**										
Cefaclor	95.1	3.4	1.6	0.27	/	99.7	S→R	No efficacy	Other cephalosporin/amoxicillin-clavulanate	
(7536)	(7163)	(253)	(120)	(21)		(7515)				
Cefuroxime-axetil	98.7	1.1	0.1	1.2	97.5	1.3	S→I/R	Reduced or no efficacy	Other cephalosporin/amoxicillin-clavulanate	
(15381)	(15183)	(175)	(23)	(189)	(14994)	(198)				

***S. pneumoniae*** CLSI 2012 intermediate breakpoint (mg/L) amoxicillin-clavulanate 4/2, cefaclor 2, ofloxacin 4; EUCAST 2012 intermediate breakpoint (mg/L) amoxicillin-clavulanate 1–2, cefaclor 0.06-0.5, ofloxacin 0.25-4; CLSI 2012 resistance breakpoint (mg/L) amoxicillin-clavulanate ≥8/4, cefaclor ≥4, ofloxacin ≥8; EUCAST 2012 resistance breakpoint (mg/L) amoxicillin-clavulanate >2, cefaclor >0.5, ofloxacin >4.

***H. influenzae*** CLSI 2012 intermediate breakpoint (mg/L) cefaclor 16, cefuroxime axetil 8, cefuroxime parenteral 8, azithromycin ND, clarithromycin 16, telithromycin 4; EUCAST 2012 intermediate breakpoint (mg/L) cefaclor ND, cefuroxime axetil 0.25-1, cefuroxime parenteral 2, azithromycin 0.25-4, clarithromycin 2–32, telithromycin 0.25-8; CLSI 2012 resistance breakpoint (mg/L) cefaclor ≥32, cefuroxime axetil ≥16, cefuroxime parenteral ≥16, azithromycin ND, clarithromycin ≥32, telithromycin ≥8; EUCAST 2012 resistance breakpoint (mg/L) cefaclor >0.5, cefuroxime axetil >1, cefuroxime parenteral >2, azithromycin >4, clarithromycin >32, telithromycin >8.

***M. catarrhalis*** CLSI 2012 intermediate breakpoint (mg/L) cefaclor 16, cefuroxime axetil 8; EUCAST 2012 intermediate breakpoint (mg/L) cefaclor ND, cefuroxime axetil 0.25-4.

CLSI 2012 resistance breakpoint (mg/L) cefaclor ≥32, cefuroxime axetil ≥16; EUCAST 2012 resistance breakpoint (mg/L) cefaclor >0.12, cefuroxime axetil >4.

## Discussion

During last two decades the emergence and spread of resistance to several antimicrobial agents in the most common respiratory pathogens has been observed worldwide [14-16].

Classification of susceptibility/resistance depends on the breakpoints that are used routinely in the clinical laboratory and that influence clinical decision-making. However, these breakpoints vary over time and due to differing guidelines. Hence, the importance of maintaining a database of raw MIC values rather than categorical reports from laboratories to track resistance trends.

CLSI, the most widely used guideline, has based preliminary breakpoints on MIC distributions, pharmacokinetic–pharmacodynamic (PK–PD) parameters and mechanisms of antimicrobial resistance. These suggestions were later confirmed in clinical trials [17]. Modern principles and methodologies are now utilized to evaluate the PK–PD of antimicrobials. EUCAST uses PK–PD simulations as a chief component of its breakpoint-setting process for old and new antimicrobials [18,19].

Due to these differences, CLSI and EUCAST breakpoints are widely divergent in several instances and the adoption of CLSI or EUCAST interpretive criteria may therefore lead to different results and conclusions.

In this study, we have compared the susceptibility data calculated using both EUCAST and CLSI breakpoints for large numbers of respiratory pathogens collected during several national and international studies to discuss the implications, if any, for empiric therapy of patients to be treated for community-acquired respiratory infections.

Concerning *S. pyogenes*, a number of antibiotics have been shown to be effective in treating group A streptococcal pharyngitis. Penicillin, however, remains the treatment of choice because of its proven efficacy and safety, and its narrow spectrum and low cost [4].

To date no strain of penicillin-resistant *S. pyogenes* has been described worldwide and the adoption of the EUCAST susceptibility breakpoint (0.25 mg/L) which is even less restrictive than CLSI (0.12 mg/L) does not change the present scenario.

Macrolides are a suitable alternative for patients allergic to penicillin. However, macrolides have often been incorrectly used as first-line agents, leading to high rates of macrolide resistance [20]. For the patient infected with an erythromycin-resistant strain of *S. pyogenes* and unable to tolerate β-lactam antibiotics, clindamycin is an appropriate alternative, in those countries where cross-resistance involving macrolides, lincosamides and streptogramins is not widespread. Using EUCAST breakpoints, the percentages of macrolides and clindamycin susceptible isolates were very similar to those obtained following CLSI criteria and no relevant discrepancies were observed.

Fluoroquinolone-resistant *S. pyogenes* strains are becoming a common finding both in adult and paediatric patients [21-23] and the adoption of EUCAST breakpoints, more restrictive than CLSI for levofloxacin, can contribute to further increase the rates of levofloxacin-resistant strains observed. However, respiratory fluoroquinolones are not yet recommended for respiratory infections due to *S. pyogenes* and, at present, replacing CLSI with EUCAST breakpoints can produce relevant changes only for epidemiological studies.

During the last two decades, surveillance studies continued to reveal increasing resistance of *S. pneumoniae,* the leading cause of pneumonia, otitis media and rhinosinusitis, to a variety of antimicrobial agents, including first line agents beta-lactams, macrolides, and quinolones [14-16].

EUCAST and CLSI benzylpenicillin breakpoints for *S. pneumoniae* are both in relation to the dosage. Comparing breakpoints referring to a similar dosage (2.4 gm 4 times or 1.2 gm 6 times daily for EUCAST and 2 million units 6 times daily for CLSI) EUCAST susceptibility breakpoint (≤1 mg/L) was more restrictive than CLSI breakpoint (≤2 mg/L) and higher dosages are suggested by EUCAST (2.4 gm 6 times daily) to cope with pneumococcal strains displaying a benzylpenicillin MIC of 2 mg/L. However, it has been shown that there is no relationship between mortality and penicillin MIC ≥ 2 mg/L and the CLSI suggest that intermediate strains (4 mg/L) may require penicillin dosages of 18 to 24 million units [24].

In contrast to CLSI, EUCAST did not publish specific breakpoints for amoxicillin or amoxicillin-clavulanate, the first line agents for otitis media and rhinosinusitis. For both agencies, isolates fully susceptible to benzylpenicillin can be reported as susceptible to amoxicillin (with or without a beta-lactamase inhibitor), otherwise EUCAST suggests using ampicillin to categorize susceptibility to amoxicillin and amoxicillin-clavulanate.

Most MIC values for penicillin, ampicillin and amoxicillin differ by no more than one dilution step and several studies have shown that amoxicillin MICs are lower than the MICs of penicillin and/or ampicillin [9]. Since the EUCAST ampicillin breakpoint for susceptibility is ≤ 0.5 mg/L and the CLSI breakpoint for amoxicillin is ≤2 mg/L, the EUCAST breakpoint is once again more restrictive than the CLSI. In those countries, such as Italy, where CLSI criteria will be replaced by EUCAST criteria, higher rates of penicillin and amoxicillin resistance are to be expected. The more restrictive EUCAST approach, on the one hand reduces the risk of discordant therapy, but on the other hand it could have a detrimental ecological consequence due to use of alternative agents such as fluoroquinolones, further increasing the burden of antimicrobial selective pressure.

Concerning the other beta-lactams, the most divergent results were seen with cefaclor. Based on PK/PD considerations, the EUCAST breakpoints for cefaclor (0.032/0.5 mg/L) have been set to ensure that the wild type is reported intermediate, indicating the need for high dosage to treat infections with wild type isolates, and any isolates with raised MICs are considered resistant.

The adoption of EUCAST breakpoints for *S. pneumoniae* has limited or no impact on the activity of macrolides and fluoroquinolones, with the exception of ofloxacin. The EUCAST breakpoints for ofloxacin (0.125/4 mg/L) have been set to ensure that the wild type is reported intermediate, indicating the need for high dosage to treat infections with wild type isolates, and any isolates with raised MICs are considered resistant. Ciprofloxacin and ofloxacin (being first on the market) were approved for pneumococcal infections. However, since respiratory fluoroquinolones with enhanced activity against *S. pneumoniae* (levofloxacin, moxifloxacin or gemifloxacin) have become available, they have replaced second generation fluoroquinolones (ofloxacin or ciprofloxacin) in the guidelines [5,6].

*M. catarrhalis* and *H. influenzae* are implicated in a significant proportion of cases of acute exacerbations of chronic bronchitis, acute bacterial otitis, acute bacterial rhinosinusitis and seem to also have a role in community-acquired pneumonia [25,26]. The main resistance problem related to these two pathogens is the production of beta-lactamase (penicillinase). Rates for penicillinase producers reaches >80% in *M. catarrhalis* worldwide, and varies considerably with the geographic area for *H. influenzae*[15], but with the exception of penicllin and aminopenicillins, other molecules retain a good activity against these species. EUCAST ampicillin-sulbactam, amoxicillin-clavulanate and cefuroxime (parenteral use) breakpoints are lower than those suggested by CLSI and higher rates are expected following the introduction of EUCAST breakpoints. EUCAST breakpoints for cefuroxime axetil and cefaclor, based on PK/PD breakpoints, are 4- and 5-fold dilution lower than those of CLSI respectively and this shift translated into major and very major discrepancies when percentages of susceptible strains were calculated using the two criteria. According to CLSI breakpoints 98% and 99% of *M. catarrhalis* and *H. influenzae* were susceptible to cefuroxime axetil respectively, while with the adoption of EUCAST breakpoints the majority of the strains (>98%) were categorized as intermediate or resistant. A similar shift was observed for *H. influenzae, M. catarrhalis* and cefaclor.

The adoption of EUCAST criteria for *H. influenzae* produced very major discrepancies for macrolides when the rates of susceptibility calculated using EUCAST and CLSI breakpoints were compared, as breakpoints for macrolides and related antibiotics have been set by EUCAST to categorize wild type *H. influenzae* as intermediate. The impact of this discrepancy should be limited on empirical therapy, since it is already well known that the correlation between macrolide MICs and clinical outcome is weak due to pharmacokinetic limitations [27].

*S. aureus* causes community-acquired respiratory tract infections less frequently than *S. pneumoniaeH. influenzae* and *M. catarrhalis*, but it is particularly implicated in community-acquired infections in elderly patients [28]. Methicillin-resistant *S. aureus* (MRSA) can be resistant to multiple antimicrobials compromising the utility of many currently licensed antimicrobials. The replacement of CLSI with EUCAST breakpoints did not produce relevant changes for all the comparable antibiotics, including oxacillin and cefoxitin, thus no difference in MRSA rates are to be expected.

While the limited discrepancies observed for *S. pyogenes* are not expected to have a clinical impact on the therapeutic choice for pharyngotonsillitis, higher rates of penicillin, amoxicillin and amoxicillin-clavualante resistance in *S. pneumoniae* could translate in increasing dosages of these agents or increasing use of alternative drugs as suggested by national and international guidelines to treat respiratory tract infections.

Cefaclor and/or cefuroxime axetil, when adopting EUCAST breakpoints, have reduced in vitro activity against *S. pneumoniae*, *H. influenzae* and *M. catarrhalis* in comparison to CLSI.

This observation could lead to promote further clinical studies to better define the role of these antibiotics and/or to the revision of those guidelines suggesting these molecules as alternative antimicrobial agents for upper respiratory tract infections both in adults and paediatric patients [29-33].

A possible limitation of our study is the heterogeneous source of the MIC data. However, we analysed a very large number of strains and the discrepancies highlighted here (only major and very major) are likely the result of EUCAST breakpoint changes, rendering all wild-type microorganisms intermediate or resistant to the specific antibiotic studied.

In Countries, where EUCAST breakpoints are going to be adopted, clinicians should be aware that they could be faced with antibiotic-resistant respiratory pathogens more frequently than before. However, the susceptibility results provided by the microbiology laboratory, according to the EUCAST Committee should be more consistent with pharmacokinetic–pharmacodynamic data and clinical evidence reported in literature.

If the CLSI revises it breakpoints, making them as restrictive as those suggested by EUCAST, the changes highlighted in the present work and the possible revision of guidelines for the management of respiratory tract infections will also be expected in those countries adopting CLSI interpretive criteria.

## Summary

· In European countries CLSI breakpoints are going to be replaced with those published by EUCAST.

· As there are many differences between the CLSI and EUCAST breakpoints, we discuss the impact of this replacement on the overall susceptibility and the possible consequences occurring in clinical practice.

· With rare exception, for the majority of pathogen/antibiotic combinations the impact is limited.

· For these exceptions, we recommend that further clinical studies are promoted to better define the clinical role of some antibiotics.

## Abbreviations

CLSI: Clinical and Laboratory Standards Institute; EUCAST: European Committee on Antimicrobial Susceptibility Testing; MIC: Minimal inhibitory concentration; PK-PD: Pharmacokinetic-pharmacodynamic; MRSA: Methicillin-resistant *S. aureus*.

## Competing interests

The authors declare that they have no competing interests.

## Authors’ contributions

AM designed the study, interpreted the data and wrote the article, SE contributed to write the clinical discussion, RB acquisition of microbiological data, MB contributed to write the clinical discussion, ED contributed to design the study, revised the microbiological data and the manuscript. All authors read and approved the final version for publication.

## Authors’ information

Anna Marchese is Associate Professor of Clinical Microbiology at the University of Genoa, Genoa, Italy. Her research fields include: epidemiology of mechanisms of antibiotic resistance, antimicrobial susceptibility testing, antimicrobial profile of new drugs, bacterial genetics. She acts as referee for several international journals. She is a member of the editorial Board of the Journal of Chemotherapy. She has published more than 80 international papers.

Susanna Esposito is Associate Professor of Pediatrics, University of Milan, Milan, Italy. She is an Editor of BMC Infectious Diseases and a Board Member of the European Society of Paediatric Infectious diseases. She is the head of the Paediatric Infectious Diseases Unit of the Fondazione IRCCS “Ospedale Maggiore Policlinico, Mangiagalli e Regina Elena”, Milano, Italy.

Ramona Barbieri is a Microbiology PhD student working at the Microbiology Unit, DISC, University of Genoa, italy.

Matteo Bassetti pursued his medical education at the Yale University School of Medicine, New Haven, CT, USA with an Infectious Diseases (ID) fellowship, and gained a PhD in Solid Organ Transplantation and Infectious Diseases at the University of Genoa School of Medicine. Dr Bassetti has worked as coordinator of ID consultation team and member of the Infection Control and Hospital Epidemiology in San Martino Teaching Hospital in Genova, Italy from 2001 to 2011.

Dr. Bassetti is currently Head of the Infectious Disease Division of the Santa Maria Misericordia University Hospital in Udine, Italy. Dr Bassetti is Professor of Infectious Diseases in the Postgraduate School of Infectious Diseases, Tropical Diseases, Hematology and Public Health of the University of Genoa, School of Medicine.

Professor Eugenio A. Debbia is Full Professor of Clinical microbiology at the University of Genoa, Genoa, Italy. His research fields include: epidemiology of mechanisms of antibiotic resistance, antimicrobial susceptibility testing, antimicrobial profile of new drugs and bacterial genetics. He is the Editor in chief of the official journal of the Italian Association of Clinical Microbiologists.

## Pre-publication history

The pre-publication history for this paper can be accessed here:

http://www.biomedcentral.com/1471-2334/12/181/prepub
